# The Influence of Health Messages in Nudging Consumption of Whole Grain Pasta

**DOI:** 10.3390/nu11122993

**Published:** 2019-12-06

**Authors:** Giovanni Sogari, Jie Li, Michele Lefebvre, Davide Menozzi, Nicoletta Pellegrini, Martina Cirelli, Miguel I. Gómez, Cristina Mora

**Affiliations:** 1 Department of Food and Drug, University of Parma, 43124 Parma, Italy; davide.menozzi@unipr.it (D.M.); nicoletta.pellegrini@unipr.it (N.P.); martina.cirelli@studenti.unipr.it (M.C.); cristina.mora@unipr.it (C.M.); 2 Charles H. Dyson School of Applied Economics and Management, Cornell University, Ithaca, NY 14850, USA, jl2522@cornell.edu (J.L.); mig7@cornell.edu (M.I.G.); 3 White Lodging School of Hospitality & Tourism Management, Purdue University Northwest, Hammond, IN 46323, USA; mlefebvre@pnw.edu

**Keywords:** choice architecture, dining environment, campus, college students, cereal grains, dietary fiber, vitamins, claim, information

## Abstract

Health messages may be an important predictor in the selection of healthier food choices among young adults. The primary objective of our study is to test the impact of labeling whole grain pasta with a health message descriptor displayed at the point-of-purchase (POP) on consumer choice in a campus dining setting. The study was conducted in a large US college dining venue during lunch service; data were collected during a nine-week period, for a total of 18 days of observation. Each day, an information treatment (i.e., no-message condition; vitamin message; fiber message) was alternated assigned to whole grain penne. Over the study period, the selection of four pasta options (white penne, whole grain penne, spinach fettuccine, and tortellini) were recorded and compiled for analysis. Logistic regression and pairwise comparison analyses were performed to estimate the impact of health messages on diners’ decisions to choose whole grain penne among the four pasta types. Our results indicate that only the message about vitamin benefits had a significant effect on this choice, with a 7.4% higher probability of selecting this pasta than the no-message condition and 6.0% higher than the fiber message condition. These findings suggest that psychological health claims (e.g., reduction of fatigue) of whole grains seem more attractive than physiological health claims (e.g., maintaining a healthy weight) for university students. In line with the 2015–2020 Dietary Guidelines for Americans, our results suggest that small changes made at the POP have the potential to contribute to significant improvements in diet (e.g., achieving recommended levels of dietary fiber). These findings have important implications for food service practitioners in delivering information with the greatest impact on healthy food choices.

## 1. Introduction

The move of young adults from living at home with their parents to college life is considered a critical phase for healthy eating behavior and can contribute to altered dietary habits such as high-energy intake and non-nutritious, unbalanced diets [1,2,3]. This change of life can contribute to the development of noncommunicable diet-related diseases (e.g., obesity, type 2 diabetes, some types of cancer, and cardiovascular diseases) in later stages of life [4,5]. 

In response, the U.S Department of Agriculture (USDA), has called for a transition toward healthier diets, including foods rich in nutrients (e.g., essential vitamins and minerals as well as dietary fiber (“Nondigestible soluble and insoluble carbohydrates (with three or more monomeric units), and lignin that are intrinsic and intact in plants; isolated or synthetic nondigestible carbohydrates (with three or more monomeric units) determined by FDA to have physiological effects that are beneficial to human health” [6]) that contribute to positive health outcomes [7]. 

According to health and nutrition experts, diets with a significant intake of whole grains (i.e., “Grains and grain products made from the entire grain seed, usually called the kernel, which consists of the bran, germ, and endosperm. If the kernel has been cracked, crushed, or flaked, it must retain the same relative proportions of bran, germ, and endosperm as the original grain in order to be called whole grain. Many, but not all, whole grains are also sources of dietary fiber.” [7] (p. 96)) and low intake of refined grains are associated with reduced risks of chronic disease [8,9] and are associated with increased diet quality [10,11]. Specifically, thanks to the health-promoting components of whole grains, such as dietary fibers, vitamins, minerals, antioxidants, and other bioactive compounds [12,13,14], the consumption of this type of cereal grain is often associated with a lower risk of metabolic syndrome, type 2 diabetes [15], and cardiovascular diseases [16]. Moreover, whole grain intake has been associated with reduced risks of obesity and weight gain in the general population [8,9], including young adults [17]. One reason is that diets rich in dietary fibers and whole grains tend to have larger food volume, which results in increasing the status of satiety [18], therefore reducing hunger [8]. This increased satiety and reduction in hunger lead to lower calorie intake, resulting in weight loss or weight gain prevention.

In addition, whole grains are also rich sources of vitamins and minerals [7]; generally accepted scientific evidence suggests that these nutrients, especially B vitamins (thiamin, riboflavin, niacin, folic acid), iron, and magnesium contribute to the reduction of tiredness and fatigue [19].

In our study, we use health claims, including messages with better-known whole grain benefits (e.g., fibers have positive effects on weight management) as well as those with less familiar and more specific claims (e.g., the relationship between vitamins and reduced fatigue) [20]. It should be noted that in the US and the European Union, a food-related health claim must be approved by the FDA (the Food and Drug Administration, of the United States Department of Health and Human Services,) and EFSA (the European Food Safety Authority of the European Union), respectively, and it must be supported by a significant body of research showing the relationship between the product and the health claim [21,22,23,24]. 

Past evidence showed that on-site nutrition policies (e.g., information campaigns) can improve dietary intake, resulting in favorable effects on weight-related outcomes [7]. However, awareness and use of nutrition labels among US university students are still not well understood and are highly dependent on the type of message considered [25].

The primary aim of our study is to test the impact of labeling whole grain pasta with a health message displayed at the point-of-purchase (POP) in a US university campus dining setting on diners’ choice of whole grain penne among four types of pasta. We hypothesize that a health message increased the probability of selecting the whole grain pasta relative to the no-message condition. A secondary aim is to determine which message intervention (physiological vs. psychological health benefit) would have a larger effect on whole grain pasta choices.

Our paper contributes to the literature by providing insights about the effectiveness of communication interventions about health-promoting food components as a mechanism for encouraging healthy eating behavior, without reducing consumer choice. 

## 2. Background

A wide variety of interventions have been utilized to encourage healthy food choices, including altered choice environments to improve selections of desired options (i.e., nudging). Thaler & Sunstein [26] define a nudge as “any aspect of the choice architecture that alters people’s behavior in a predictable way without forbidding any options or significantly changing their economic incentives”. The choice architecture includes all the physical elements in a defined environment that individuals experience when they make choices, including the information provided [27,28].

A systematic review by Vecchio & Cavallo [29] demonstrated that many physical elements have been taken into consideration in experiments targeting individuals, including product placement, the effect of priming, environmental cues, and varying portion sizes. Here, we focus on studies that have used point-of-purchase (POP) information to show the effect of different types of POP messaging on food choices among university students. First, studies have shown that at the POP rational decision behavior is affected by heuristic judgment [28]. For instance, Peterson et al. [30] showed positive changes in students’ perceptions and selections of healthy foods by introducing a three-week point-of-selection intervention using “benefit-based messages” in a dining setting. Other studies [31,32] showed how information placement (e.g., 4 × 2.5 in signs placed next to the targeted food), graphics (e.g., eye-catching promotion), and message content (e.g., appeal, relevance and length of the text) are important in influencing healthy food choices. As reported by a systematic review of food environment interventions in university settings [33], useful interventions are mostly focused on nutrition messages and nutrient labeling at the POP. 

Only a few studies have investigated potential interventions to specifically promote whole grain products [34,35]. In general, grains, including whole grains, are staple foods in many countries [13] and can be found in single foods (e.g., rice, oatmeal), or as an ingredient in many food products (e.g., bread, cereals, crackers, and pasta) [7]. In our study, we focused on whole grain pasta. The reasons for choosing pasta are its popularity and versatility, which make it one of the most ubiquitous durum wheat-derived products around the world [36,37]. Moreover, pasta is a food item that can be fully produced using whole grains [37]. Finally, previous studies (e.g., Larson et al. [38]) noted that the potential to influence whole-grain intake in a school environment merits investigation. 

Few US campus dining venues have promoted whole grains on the menu. However, in recent years, this trend is changing since a campus dining initiative called “Menus of Change: The Business of Healthy, Sustainable, and Delicious Food Choices” was founded in 2012 [39]. The primary focus of this initiative is to achieve healthy and sustainable menus, including the promotion of the use of whole grains across all menus on college campuses. From Menus of Change, the Menus of Change University Research Collaborative (MCURC) was founded jointly by Stanford University and The Culinary Institute of America. MCURC was established with working groups of scholars and campus dining leaders interested in using college and university dining as a platform to establish and accelerate efforts to move campus diners towards healthier and more sustainable menus. One of the working groups’ priorities is on “menuing healthy”. The primary goal is to use campus dining venues as a platform for education and learning around these principles, and thus educate students and prepare them for a future of healthy and sustainable food decisions. Some of the core principles, in line with the USDA’s 2015–2020 Dietary Guidelines for Americans, include making whole, intact grains the new norm, and focusing on whole, minimally processed foods; both of which focus on incorporating more whole grains into the diet. Although the Dietary Guidelines for Americans states that at least half of total grain intake should be provided by whole grains [6], most of the population today still fail to meet the recommended levels of whole grain intake [9,10,19]. In our study, the nudge was implemented in order to draw attention to whole grain penne, which was considered the relatively healthier item among different pasta options in the focal dining venue. The rationale was to inform individuals of the nature and consequences of their choices [40].

## 3. Materials and Methods

### 3.1. Data Collection 

The study was conducted in a large US college dining venue during lunch (11 am–2 pm), from February to April 2019. The Institutional Review Board (IRB) of the Office of Research Integrity and Assurance of Cornell University approved this study (Protocol ID: 1810008359) and waived informed consent because no identifying information was collected from participants. Data were collected two weekdays per nine weeks (*n* = 18 days of observation in total) in an ‘a la carte’ college dining setting where students could choose foods from salad, soup, grill, Mexican, Asian, and pasta stations. 

The experiment employed different nudges to promote the purchase of whole grain pasta. The “health claim” nudge informs individuals of the nature and health consequences of their food choices. The field experiment was designed over a nine-week period, as seen in Table 1, such that: (1) Weeks 1, 4, and 7 were a no-message condition weeks (control group) where no nudges were implemented; and (2) Weeks 2, 3, 5, 6, 8, and 9 were the experimental nudge weeks in which the two different treatments were alternately implemented to the whole grain penne dish (i.e., one day the vitamin message and the other day the fiber message, and vice versa). 

To achieve the highest level of accuracy, the design was balanced (i.e., each of the treatments occurs the same number of times in each period). Based on previous literature [41], two types of messages were developed: (1) a health claim referring to the function of the body (e.g., increase the sense of satiety) and (2) a health claim referring to psychological functions (e.g., the reduction of tiredness). The content of these messages was based on the higher content in fiber and niacin (vitamin B3) in whole grain wheat flour than in refined grain [42], which is present in whole grain pasta [43]. Specifically, we considered the latest scientific opinions on the substantiation of health claims related to wheat bran fiber and increase in faecal bulk [44], and niacin for reduction of tiredness and fatigue, in situations of inadequate micronutrient status [13,20]. Currently, the claims used in our study are approved by EFSA and not by the FDA. 

See Table 2 for the complete information provided.

Each day of data collection, two research assistants discretely recorded the number of diners (i.e., students) who selected one of the four pasta options served (white penne, whole grain penne, spinach fettuccine, and tortellini). Customers were given the option to first choose the type of (plain) pasta, then the sauces, and toppings, and finally cheese. Thereby, we assumed that the type pasta (e.g., white vs. whole grain penne) is the main factor influencing the selection because all other choices are made afterwards. As a result, the attribute of taste, which is often more important than health claims in influencing purchasing decisions [45], is taken into consideration only after choosing the type of pasta. It was not possible to control for other determinants of food choices, such as more physiological mechanisms (e.g., hunger and satiety) and habitual patterns (e.g., past choices) [28].

Moreover, no changes were made to how the pasta was prepared or served over the period of data collection. The price for all types of pasta was identical and thus cannot be considered a limiting factor in the choice of pasta. Lastly, no other alterations were made to the pasta menu signage; flavor of sauces, pasta, or toppings was not highlighted in any way to keep the offerings consistent across treatments.

Table 3 shows the conceptual level of our nudging study. 

### 3.2. Data Analysis

The data were first recorded in Excel (Microsoft Excel, 2017) using a mobile device at the dining venue. Analyses were carried out using the Statistical Package for Social Sciences (SPSS) version 24 SPSS (SPSS Inc., Chicago, IL, USA) and STATA version 16 (Stata Corporation, College Station, TX, USA).

Data were coded using numbers to represent the different types of pasta (i.e., ‘white penne’ = 1; ‘whole grain penne’ = 2; ‘tortellini’ = 3; and ‘fettuccine’ = 4), and various types of health message treatments (i.e., “no-health message control group” = 0; “rich in vitamins” = 1; “rich in fiber” = 2). The only moderator explored was gender because it was the only sociodemographic characteristic easily recognized without interacting with subjects in our sample. To investigate the association between the whole grain pasta choice probability and various health message treatments, a logistic regression was used, including treatment and gender as predictors. 

Specifically, we employed a generalized linear model with a binomial distribution in a logit link function with post hoc analysis from that model to compare the different probabilities across treatments. The binary dependent variable in the logistic regression model was set to one if whole grain penne was chosen and zero otherwise. The week and days when of data collection were included as fixed effects in the statistical analysis to control of time effects on the pasta choices.

## 4. Results

First, most diners chose tortellini (*n* = 1325) and spinach fettuccine (*n* = 992), followed by white and whole grain penne. Specifically, 725 and 692 of 3734 total diners selected the white penne (19.4%) and the whole grain penne (18.5%), respectively. 

**Figure 1:**
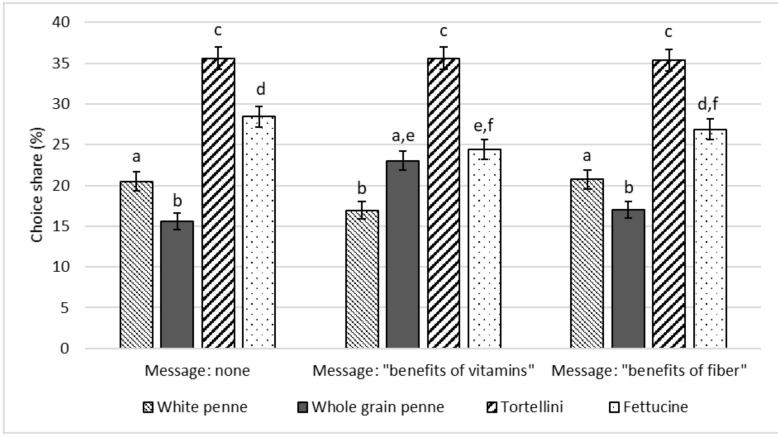
Diners choosing white penne, whole grain penne, tortellini, and fettuccine on the total, by treatment (% values, error bars represent standard errors). Different letters imply values significantly different at *p* < 0.05 probability level between products within the same treatment (Wilcoxon signed rank sum test) and between different treatments (Wilcoxon–Mann–Whitney test).

Figure 1 presents the percentage shares of diners choosing different types of pasta across the three treatment groups. The Wilcoxon nonparametric paired tests show that labelling whole grain penne with the vitamin message resulted in a 7.4% higher probability of selecting this pasta than the no-message condition (*p* < 0.001) and 6% higher than the fiber message condition (*p* < 0.001). No significant difference was observed between the fiber message and the selection of whole grain penne. The results also show that when presented with the vitamin message for whole grain penne, the percentage of diners who chose spinach fettuccine decreased by 4% relative to the percentage of diners who chose spinach fettucine when no message was presented (*p* < 0.05). The probability of choosing spinach fettuccine is not significantly different than choosing whole grain penne when presented with the vitamin message (*p* = 0.482). These effects were not observed for tortellini pasta, with its frequency remaining stable across treatments. 

Table 4 shows the results of the logistic regression, only considering whole grain penne within the total of individuals who chose penne, because white and whole grain penne can be considered close substitutes to each other. The results show that the coefficient for the vitamin message is positive and significant, indicating that the presence of the vitamin message about the whole grain pasta increases the probability that diners chose whole grain penne relative to the control situation (no-message condition). Moreover, the results show that the coefficient of the variable Female is not significant at 5%, indicating that gender has no significant impact on diner’s probability in choosing whole grain penne. Finally, the significance of the constant term indicates that ceteris paribus, the choice probability of whole grain penne is lower than for white penne.

We also tested the interactions of gender and the different health messages, but did not find any significant interaction effect. This result suggests that males and females respond to information treatments in a similar way.

To further test the impact of information on whole grain penne relative to all other types of pasta, we employed another logit regression considering all pasta types. In this case, the dependent variable was equal to 1 if diners chose whole grain penne, 0 if they chose one of the remaining three types (white penne, spinach fettuccine or tortellini). The results, presented in Table 5, show that the choice probability of whole grain penne is significantly lower than for the other pasta types (constant term significant at *p* < 0.001). However, the choice probability of whole grain penne is higher in the presence of the vitamin message relative to other pasta types. No significant difference was observed with the information treatment about fiber. Considering all pasta types, the gender effect is not significant; in other words, when all pasta types are considered, females and males have the same probability of choosing whole grain penne.

## 5. Discussion

This paper provides evidence that placing a health message about vitamin benefits (i.e., rich in B vitamins, which help to reduce fatigue) related to whole grain penne in proximity to the targeted pasta significantly increased the number of individuals choosing this option versus white penne. The frequency of the selection of spinach fettuccine decreased when the whole grain penne was labeled with the vitamin message. This suggests that “reduce fatigue” information may change preferences because whole grain penne is perceived to have an added value compared to spinach fettuccine in this context. Nevertheless, no changes in selection frequency were found for the tortellini, which remained constant even when the whole grain penne was labelled with a health message. This result indicates that the type of pasta is the first factor influencing the decision process, especially when it includes other ingredients (e.g., filled pasta such as tortellini). This confirms that palatability of foods is one of the main determinants of food choice [28]. However, our results suggest that connecting the pleasure of eating with healthy food attributes is a good approach [46]. 

In general, even if whole grain products are generally perceived as healthier [12], the lack of an effective health message campaign promoting these benefits results in low levels of consumer attention and interest towards whole grains [7]. One possible explanation could be that consumers may not understand the health benefits of whole grains and/or may not know how to identify labels and interpret recommendations of such products [20,21]. 

Therefore, simply making a statement on the label of a cereal grain product such as ‘100% whole grain’ may not be as effective as presenting health-related benefits to influence consumer choice. As suggested from previous studies [47,48], one of the potential barriers to purchase whole grain products is the low knowledge and awareness of their health benefits. Although most people are aware that whole grains are healthy and less processed compared to refined grains [49], there is only a basic understanding of which are the specific health outcomes. 

In fact, our results suggest that the presence of only the mention of ‘whole grain’ is not strong enough to influence student’s choice towards whole grain pasta. This could mean that consumers did not associate healthy benefits at the moment of selection, resulting in a lower motivation to choose whole grain penne. Therefore, information about health outcomes from eating whole grains may aid in increasing the likelihood of selecting this pasta over regular types [8].

Even if previous studies (e.g., Marquar et al. [50]) report that individuals usually associated increased fiber intake with the main health benefits of eating whole-grain foods, our results suggest that other health-promoting components (i.e., B vitamins that help to reduce fatigue) have a greater impact in influencing the consumption of whole grains. Our findings also suggest how psychological health claims of whole grains seem more attractive than physiological health claims for university students. Several possible explanations could be drawn. First, the target population might be more interested in “scholarly” benefits, such as the reduction of fatigue, rather than weight management. Nonetheless, it is likely that the information provided will be more effective when it addresses specific needs to the target population [51]. Moreover, when a decision-making process occurs between a long-term benefit (e.g., ‘*maintain a healthy weight*’) and a short-term benefit (e.g., ‘*be more alert*’), the latter outcome is likely to be more preferred [27].

Until now, most of the nutrition information provided to consumers focused on macronutrients [52], but our results indicate that providing health benefits of micronutrients (i.e., vitamins) affects consumer food choices. This provides a possible explanation for why the claim referring to vitamins attracted the most interest from consumers [53]. In contrast with previous studies [41,54], which showed how individuals tend to rate psychological health claims as less attractive than physiological health claims, our results indicate the significant impact of psychology/behavior-based benefits (e.g., reduce fatigue). We suggest that attentional account could explain why the latter option is more preferred, while the first appear as less attractive. For instance, the attentional processing is strongly related to different contents of the health benefit [55] as well as the specific topic and audience that the message is addressed to. However, the most common features that may influence persuasion is the perceived argument strength, which refers to individual perceptions of the quality and persuasiveness of the message [56]. 

Therefore, one of the possible explanations for our findings could be related to how the messages were framed and their ability to evoke recipients’ attention and engage them. Perhaps the connection between fiber and satiety (‘Whole grains are rich in fiber, which will make you feel more full’) was not reported straightforwardly and the content was too vague (‘Feeling fuller will help you maintain a healthy weight’), resulting in lower ability to evoke recipients’ attention compared to specificity of the vitamin message (‘A reduction in fatigue will help you be more alert’).

There are, however, other possible explanations. For instance, the content of the vitamin B message is newer (i.e., novelty component) and perhaps this could attract the attention of consumers more. 

### 5.1. Implications

Previous studies [18,57] suggest that whole grain and fiber-rich foods are generally less appealing, but our results show that by keeping the type of pasta constant (i.e., penne), the promotion of whole grain via healthy attribute messaging increases the choice probability of whole grain pasta.

Our findings confirm how the POP nutrition information can help college students make informed and healthy choices [32,58] and could be used to plan interventions in higher education institutions, highlighting the importance of developing messages to influence food choices. For instance, when framing messages (e.g., generic vs. specific health, or benefit vs. risk communication), expert opinion needs to be taken carefully into consideration along with responsiveness from consumers. We showed how more general health benefits (e.g., maintain body weight) is less attractive than a particular outcome (e.g., be more alert). 

Consequently, nutrition and dietitian experts, food service management staff, and consumer behavior scientists should work together to develop effective and long-term targeted intervention strategies (e.g., message framing) designed for specific demographics (e.g., university students) within the campus environment (e.g., dining facilities and convenience stores) [1,27,30,31,59,60]. 

From a marketing perspective, our findings underscore the role of communicating the content and benefits of a food product in an effective and easy-to-read way (e.g., claim related to health benefits) [61]. This approach could be taken into consideration by different stakeholders, including food companies, which can use labels on products to communicate the health benefits of food to consumers [62]. 

Regulators are also a key stakeholder group which is involved in evidence-based health policy decision-making strategies to improve dietary practices [63]. Nutrition labeling policy could benefit from understanding how specific changes in communication strategies affect consumer behavior.

### 5.2. Strengths and Limitations

One of the strengths of this field study is that the experiment was carried out in a natural context (a campus dining venue), resulting in the exploration of more natural consumer behavior in response to a nudge [27].

**Figure A1:**
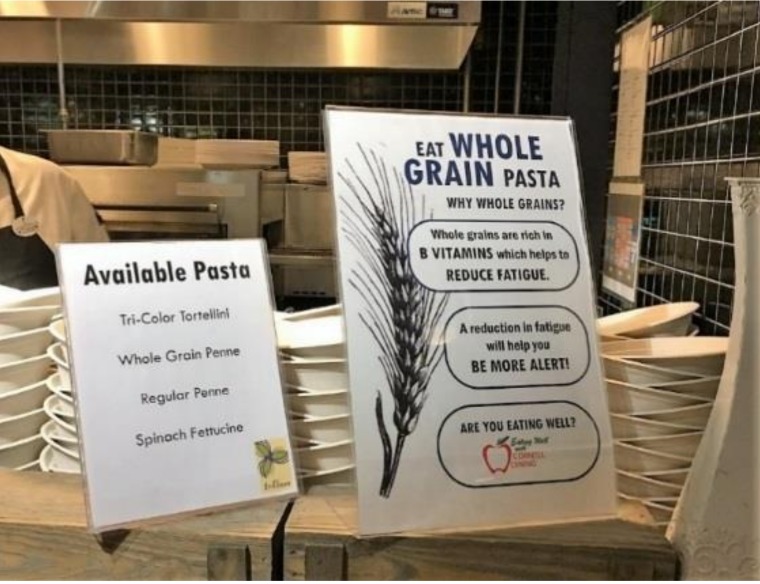
Example label. The left poster depicts the four pasta options available at the station; the right poster shows the long health message about vitamins and the reduction in fatigue.

**Figure A2:**
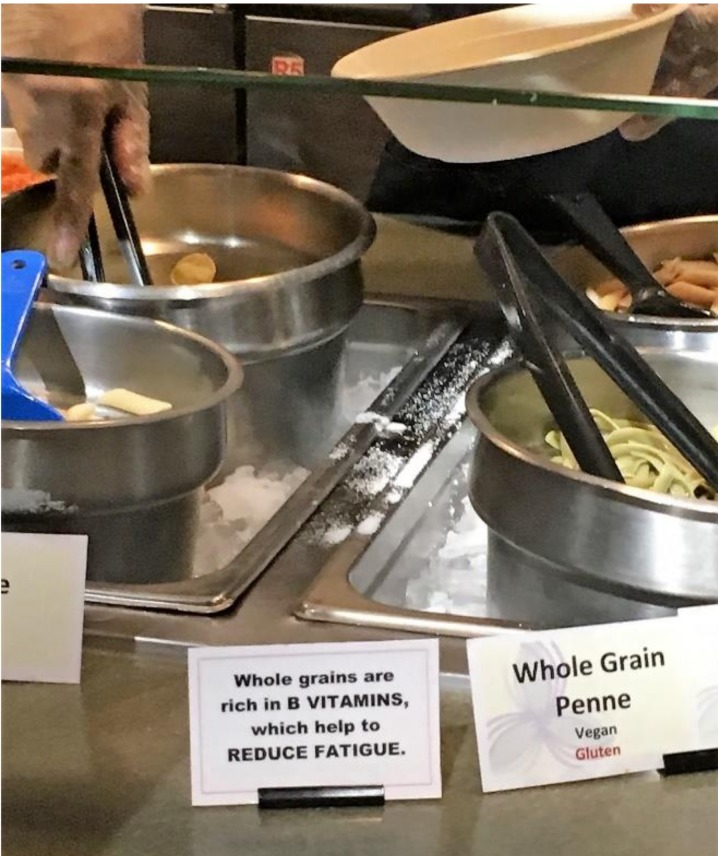
Example label. This small card depicts the short message about vitamins and the reduction in fatigue placed directly in front of the pasta.

However, this study is not without limitations. First, data collection took place only in a specific setting (college dining venue) with a specific population (university students); thus, the findings should not be generalized to other situations and target groups. Second, we did not collect any sociodemographic information from participants (except gender via observation), resulting in the risk of recurring respondents and a limited possibility to understand potential moderators of the pasta choices. The presence of a specific moderator (i.e., individual permanent trait) could modify the relationship between our dependent variables and the choice probability of whole grain penne [27]. Moreover, our study could be biased by cross-over effects from each message condition. Finally, considering that this is an observational study, we could not be certain that all subjects read the messages; our assumption is that considering the closeness of the information treatment to the pasta station (see Figure A1 and Figure A2 in Appendix A), most diners did notice the health message.

## 6. Conclusions and Future Studies

In this study, we investigated the role of health-promoting whole grain components in the selection of whole grain penne vs. other types of pasta in a college dining venue.

Our results indicate that targeted information interventions that communicate the health psychology/behavior-based benefits of consuming whole grain (i.e., reduce fatigue) at the POP increased students’ whole grain intake. 

Moreover, environmental modifications do not involve imposing eating habits and/or limiting food choices, so replacing some refined grain meals with whole grains product consumption should be easier than many other dietary changes [64]. In line with the 2015–2020 Dietary Guidelines For Americans [7], our results suggest that small changes made at the POP can contribute to significant improvements in the diet (e.g., achieving recommended levels of dietary fiber). More generally, our study confirms that it is possible to design a nudge to promote the desired behavior (e.g., eating more whole grains) without conscious effort and still let consumers have the freedom to make a choice [27]. Considering that hard regulation is often not effective to modify food consumption toward a healthy diet [28], the nudge approach could positively change eating habits in specific settings.

Our study sheds light on ways to increase consumption of whole grains, yet more research on this topic is warranted. Future research should investigate individual preferences for whole grain health claims in different eating and purchasing contexts, such as dining venues in worksites and other food service establishments (e.g., restaurants), as well as different target populations (e.g., countries where pasta is a more traditional food). We also recommend that future studies investigate the type of pasta and the healthy attributes separately by testing whole grain vs. nonwhole grain fettuccine as well as whole grain vs. nonwhole grain tortellini. 

A natural extension of this work would be to investigate how long this intervention should continue to affect preferences in the long run and create habits among consumers. Considering that the location of the message label is an important factor in the success of the intervention [31], further investigation could also focus on where to place the communication campaigns to be more effective and consider an internal validation to assess whether participants actually read the message. Further work should also take into consideration how participants’ subjectively self-reported satiety [65] could influence the choice of whole grain pasta and the impact of different health messages. 

Further investigations to address specific nutritional needs could focus on consumers’ interest in other types of pasta, such as fortified pasta with protein sources from animal or plant material [66,67]. Finally, future studies should continue to explore how to better frame the content of messages (e.g., argument quality and source credibility), explore attentional account process underlying preferences across different types of messages, and partner flavor messaging with health messaging to determine the combined impact compared to health messaging alone. 

## Figures and Tables

**Table 1:** The timeline for the study.

Week	Day 1	Day 2
1	No-message condition	No-message condition
2	Vitamin message	Fiber message
3	Vitamin message	Fiber message
4	No-message condition	No-message condition
5	Fiber message	Vitamin message
6	Fiber message	Vitamin message
7	No-message condition	No-message condition
8	Vitamin message	Fiber message
9	Vitamin message	Fiber message

**Table 2:** Health messages.

	Physiological Health Benefit	Psychological Health Benefit
Extended message provided on an 11 by 14-in poster intervention located right next to the pasta station (Figure A1 in the Appendix A).	EAT WHOLE GRAIN PASTA Whole grains are rich in fiber, which will make you feel more full. Feeling fuller will help you maintain a healthy weight!	EAT WHOLE GRAIN PASTA Whole grains are rich in B vitamins, which help to reduce fatigue. A reduction in fatigue will help you be more alert!
Short message provided in 3 by 2-in card intervention placed directly in front of the targeted pasta (Figure A2 in the Appendix A).	Whole grains are rich in fiber, which will make you feel more full.	Whole grains are rich in B vitamins, which help to reduce fatigue.

Note: The brand of pasta types served at the college dining venues was Barilla (Ames, IA and Avon, NY). The nutrition composition and fact can be retrieved from its commercial web site (barilla.com/en-us/products/pasta/).

**Table 3:** Illustration of the whole grain pasta nudging study.

Elements of Nudging Research	
**Type of study**	Proof of concept (controlled-field experiment at a college dining venue).
**Independent variable** **(nudge)**	Placement of two health-promoting component messages about whole grains located at the pasta station. A no-message treatment was included as well.
**Experimental design**	Nonrandomized study design whereby individuals were exposed to experimental condition or control condition over a nine-week period
**Dependent variable**	The number of diners who selected whole grain pasta and the other types of pasta. Data was recorded by two researchers who stood next to the pasta station
**Mediators explored**	No individual data were obtained from the participants except gender (via observation) and the selected type of pasta.
**Moderators explored**	Gender was explored

**Table 4:** Regression results of whole grain penne among the total of penne meals.

Independent Variables	Coef.	Std. Err.	z	*p*-Value	95% Conf. Interval
Constant	−0.409	0.180	−2.26	0.024 **	(−0.794 – −0.055)
Vitamin message	0.528	0.249	2.12	0.034 **	(0.039 – 1.017)
Fiber message	0.036	0.249	0.15	0.883	(−0.452 – 0.525)
Female ^a^	0.213	0.109	1.95	0.052	(−0.001 – 0.428)

^a^ Reference group for gender was male. A total of 1417 observations were used. ** indicates a significant difference at *p* < 0.05.

**Table 5:** Regression of whole grain penne among the total of pasta meals.

Independent Variables	Coef.	Std. Err.	z	*p*-Value	95% Conf. Interval
Constant	−1.771	0.144	−12.25	0.000 ***	(−2.055 – −1.488)
Vitamin message	0.497	0.195	2.55	0.011 **	(0.115 – 0.879)
Fiber message	0.137	0.196	0.70	0.485	(−247 – 0.522)
Female ^a^	0.126	0.082	1.53	0.127	(−0.036 – 0.287)

^a^ Reference group for gender was male. A total of 3734 observations were used. ** indicates a significant difference at *p* < 0.05; *** indicates a significant difference at *p* < 0.001.

## References

[1] Sogari G., Velez-Argumedo C., Gómez M., Mora C. (2018). College Students and Eating Habits: A Study Using An Ecological Model for Healthy Behavior. Nutrients.

[2] Deliens T., Van Crombruggen R., Verbruggen S., De Bourdeaudhuij I., Deforche B., Clarys P. (2016). Dietary interventions among university students: A systematic review. Appetite.

[3] Crombie A.P., Ilich J.Z., Dutton G.R., Panton L.B., Abood D.A. (2009). The freshman weight gain phenomenon revisited. Nutr. Rev..

[4] World Health Organization Obesity and Overweight. https://www.who.int/en/news-room/fact-sheets/detail/obesity-and-overweight.

[5] World Health Organization (2017). Noncommunicable Diseases: The Slow Motion Disaster.

[6] Department of Health and Human Service (2016). Food and Drug Admisistration Federal Register Volume 81, Issue 103 (May 27, 2016).

[7] U.S. Department of Health and Human Services and U.S. Department of Agriculture (2015). 2015–2020 Dietary Guidelines for Americans.

[8] Jones J.M., Engleson J. (2010). Whole Grains: Benefits and Challenges. Annu. Rev. Food Sci. Technol..

[9] Ye E.Q., Chacko S.A., Chou E.L., Kugizaki M., Liu S. (2012). Greater whole-grain intake is associated with lower risk of type 2 diabetes, cardiovascular disease, and weight gain. J. Nutr..

[10] O’Neil C.E., Nicklas T.A., Zanovec M., Cho S.S., Kleinman R. (2011). Consumption of whole grains is associated with improved diet quality and nutrient intake in children and adolescents: The National Health and Nutrition Examination Survey 1999–2004. Public Health Nutr..

[11] McGill C.R., Fulgoni V.L., Devareddy L. (2015). Ten-Year Trends in Fiber and Whole Grain Intakes and Food Sources for the United States Population: National Health and Nutrition Examination Survey 2001–2010. Nutrients.

[12] Shepherd R., Dean M., Lampila P., Arvola A., Saba A., Vassallo M., Claupein E., Winkelmann M., Lähteenmäki L. (2012). Communicating the benefits of wholegrain and functional grain products to European consumers. Trends Food Sci. Technol..

[13] European Commission Whole Grain. https://ec.europa.eu/jrc/en/health-knowledge-gateway/promotion-prevention/nutrition/whole-grain.

[14] Lattimer J.M., Haub M.D. (2010). Effects of dietary fiber and its components on metabolic health. Nutrients.

[15] De Munter J.S.L., Hu F.B., Spiegelman D., Franz M., Van Dam R.M. (2007). Whole grain, bran, and germ intake and risk of type 2 diabetes: A prospective cohort study and systematic review. PLoS Med..

[16] Anderson J.W. (2004). Whole grains and coronary heart disease: The whole kernel of truth. Am. J. Clin. Nutr..

[17] Quick V., Wall M., Larson N., Haines J., Neumark-Sztainer D. (2013). Personal, behavioral and socio-environmental predictors of overweight incidence in young adults: 10-yr longitudinal findings. Int. J. Behav. Nutr. Phys. Act..

[18] Martini D., Brusamolino A., Del Bo’ C., Laureati M., Porrini M., Riso P. (2018). Effect of fiber and protein-enriched pasta formulations on satiety-related sensations and afternoon snacking in Italian healthy female subjects. Physiol. Behav..

[19] Sadler M. (2014). 13-Authorised EU health claims for vitamins and minerals. Foods, Nutrients and Food Ingredients with Authorised Eu Health Claims.

[20] (2010). EFSA Panel on Dietetic Products, Nutrition and Allergies (NDA) Scientific Opinion on the substantiation of health claims related to niacin and reduction of tiredness and fatigue (ID 47), contribution to normal energy-yielding metabolism (ID 51), contribution to normal psychological functions (ID 55), maintenance of normal blood flow (ID 211), and maintenance of normal skin and mucous membranes (ID 4700) pursuant to Article 13 (1) of Regulation (EC) No 1924/2006. EFSA J..

[21] European Commission (2012). COMMISSION REGULATION (EU) No 432/2012 of 16 May 2012 Establishing a List of Permitted Health Claims Made on Foods, Other Than Those Referring to the Reduction of Disease Risk and to Children’s Development and Health.

[22] European Commission (2006). Regulation (EC) No 1924/2006 of the European Parliament and of the Council of 20 December 2006 on Nutrition and Health Claims Made on Foods. Off. J. Eur. Union L.

[23] Food and Drug Administration Guidance for Industry: Evidence-Based Review System for the Scientific Evaluation of Health Claims. https://www.fda.gov/regulatory-information/search-fda-guidance-documents/guidance-industry-evidence-based-review-system-scientific-evaluation-health-claims.

[24] Binns N. (2014). 1-The regulation of health claims in Europe. Foods, Nutrients and Food Ingredients with Authorised Eu Health Claims.

[25] Christoph M.J., An R., Ellison B. (2016). Correlates of nutrition label use among college students and young adults: A review. Public Health Nutr..

[26] Thaler R.H., Sunstein C.R. (2008). Nudge: Improving Decisions about Health, Wealth, and Happiness.

[27] Van Kleef E., Van Trijp H.C.M. (2018). Methodological Challenges of Research in Nudging. Methods in Consumer Research, Volume 1.

[28] Leng G., Adan R.A.H., Belot M., Brunstrom J.M., De Graaf K., Dickson S.L., Hare T., Maier S., Menzies J., Preissl H. (2017). The determinants of food choice. Proc. Nutr. Soc..

[29] Vecchio R., Cavallo C. (2019). Increasing healthy food choices through nudges: A systematic review. Food Qual. Prefer..

[30] Peterson S., Duncan D.P., Null D.B., Roth S.L., Gill L. (2010). Positive changes in perceptions and selections of healthful foods by college students after a short-term point-of-selection intervention at a dining hall. J. Am. Coll. Health.

[31] Buscher L.A., Martin K.A., Crocker S. (2001). Point-of-purchase messages framed in terms of cost, convenience, taste, and energy improve healthful snack selection in a college foodservice setting. J. Am. Diet. Assoc..

[32] Freedman M.R., Connors R. (2010). Point-of-purchase nutrition information influences food-purchasing behaviors of college students: A pilot study. J. Am. Diet. Assoc..

[33] Roy R., Kelly B., Rangan A., Allman-Farinelli M. (2015). Food Environment Interventions to Improve the Dietary Behavior of Young Adults in Tertiary Education Settings: A Systematic Literature Review. J. Acad. Nutr. Diet..

[34] De Wijk R.A., Maaskant A.J., Polet I.A., Holthuysen N.T.E., Van Kleef E., Vingerhoeds M.H. (2016). An In-Store Experiment on the Effect of Accessibility on Sales of Wholegrain and White Bread in Supermarkets. PLoS ONE.

[35] Van Kleef E., Seijdell K., Vingerhoeds M.H., De Wijk R.A., Van Trijp H.C.M. (2018). The effect of a default-based nudge on the choice of whole wheat bread. Appetite.

[36] Martini D., Ciccoritti R., Nicoletti I., Nocente F., Corradini D., D’Egidio M.G., Taddei F. (2018). From seed to cooked pasta: Influence of traditional and non-conventional transformation processes on total antioxidant capacity and phenolic acid content. Int. J. Food Sci. Nutr..

[37] Brennan C.S., Delcour J.A., Poutanen K.B.T.-F.-R. (2013). 13-Fibre-enriched and whole wheat pasta. Woodhead Publishing Series in Food Science, Technology and Nutrition.

[38] Larson N.I., Neumark-Sztainer D., Story M., Burgess-Champoux T. (2010). Whole-Grain Intake Correlates among Adolescents and Young Adults: Findings from Project EAT. J. Acad. Nutr. Diet..

[39] The Culinary Institute of America (2017). Menus of Change, 2017 Annual Report.

[40] Sunstein C. (2014). Nudging: A Very Short Guide. J. Consum. Policy.

[41] Siegrist M., Stampfli N., Kastenholz H. (2008). Consumers’ willingness to buy functional foods. The influence of carrier, benefit and trust. Appetite.

[42] Truswell A.S. (2002). Cereal grains and coronary heart disease. Eur. J. Clin. Nutr..

[43] U.S.D.A FoodData Central Agricultural Research Service. Fdc.nal.usda.gov.

[44] (2010). EFSA Panel on Dietetic Products, Nutrition and Allergies (NDA) Scientific Opinion on the substantiation of health claims related to wheat bran fibre and increase in faecal bulk (ID 3066), reduction in intestinal transit time (ID 828, 839, 3067, 4699) and contribution to the maintenance or achievement of a normal body weight (ID 829) pursuant to Article 13 (1) of Regulation (EC) No 1924/2006. EFSA J..

[45] Wills J.M., Bonsmann S., Kolka M., Grunert K.G. (2012). Symposium 2: Nutrition and health claims: Help or hindrance: European consumers and health claims: Attitudes, understanding and purchasing behaviour. Proc. Nutr. Soc..

[46] Köster E.P. (2009). Diversity in the determinants of food choice: A psychological perspective. Food Qual. Prefer..

[47] Kamar M., Evans C., Hugh-Jones S. (2016). Factors influencing adolescent whole grain intake: A theory-based qualitative study. Appetite.

[48] McMackin E., Dean M., Woodside J.V., McKinley M.C. (2013). Whole grains and health: Attitudes to whole grains against a prevailing background of increased marketing and promotion. Public Health Nutr..

[49] Burgess-Champoux T., Marquart L., Vickers Z., Reicks M. (2006). Perceptions of children, parents, and teachers regarding whole-grain foods, and implications for a school-based intervention. J. Nutr. Educ. Behav..

[50] Marquart L., Pham A.-T., Lautenschlager L., Croy M., Sobal J. (2006). Beliefs about whole-grain foods by food and nutrition professionals, health club members, and special supplemental nutrition program for women, infants, and children participants/State fair attendees. J. Am. Diet. Assoc..

[51] Verbeke W. (2005). Agriculture and the food industry in the information age. Eur. Rev. Agric. Econ..

[52] Grunert K.G., Weber P., Birringer M., Blumberg J.B., Eggersdorfer M., Frank J. (2019). Do Consumers Care About Micronutrients? A Perspective on the Possible Role of Vitamin E in the Dietary Choices of Consumers. Vitamin E in Human Health.

[53] Cavaliere A., Ricci E.C., Banterle A. (2015). Nutrition and health claims: Who is interested? An empirical analysis of consumer preferences in Italy. Food Qual. Prefer..

[54] Van Kleef E., Van Trijp H.C.M., Luning P. (2005). Functional foods: Health claim-food product compatibility and the impact of health claim framing on consumer evaluation. Appetite.

[55] Imhof M.A., Schmälzle R., Renner B., Schupp H.T. (2017). How real-life health messages engage our brains: Shared processing of effective anti-alcohol videos. Soc. Cogn. Affect. Neurosci..

[56] Zhao X., Strasser A., Cappella J.N., Lerman C., Fishbein M. (2011). A Measure of Perceived Argument Strength: Reliability and Validity. Commun. Methods Meas..

[57] Jones J.M., Reicks M., Adams J., Fulcher G., Weaver G., Kanter M., Marquart L. (2002). The Importance of Promoting a Whole Grain Foods Message. J. Am. Coll. Nutr..

[58] Yepes M.F. (2014). Mobile Tablet Menus: Attractiveness and Impact of Nutrition Labeling Formats on Millennials’ Food Choices. Cornell Hosp. Q..

[59] Menozzi D., Sogari G., Mora C. (2015). Explaining vegetable consumption among young adults: An application of the theory of planned behaviour. Nutrients.

[60] Menozzi D., Sogari G., Mora C. (2017). Understanding and modelling vegetables consumption among young adults. LWT Food Sci. Technol..

[61] Corallo A., Latino M.E., Menegoli M., Spennato A. (2019). A Survey to Discover Current Food Choice Behaviors. Sustainability.

[62] Corallo A., Latino M.E., Menegoli M., De Devitiis B., Viscecchia R. (2019). Human Factor in Food Label Design to Support Consumer Healthcare and Safety: A Systematic Literature Review. Sustainability.

[63] World Health Organization Healthy Diet. https://www.who.int/news-room/fact-sheets/detail/healthy-diet.

[64] Marquart L., Wiemer K.L., Jones J.M., Jacob B. (2003). Whole grain health claims in the USA and other efforts to increase whole-grain consumption. Proc. Nutr. Soc..

[65] Ding F., Hamid N., Shepherd D., Kantono K. (2019). How is Satiety Affected When Consuming Food While Working on A Computer?. Nutrients.

[66] Fuad T., Prabhasankar P. (2010). Role of Ingredients in Pasta Product Quality: A Review on Recent Developments. Crit. Rev. Food Sci. Nutr..

[67] Liu T., Hamid N., Kantono K., Pereira L., Farouk M.M., Knowles S.O. (2016). Effects of meat addition on pasta structure, nutrition and in vitro digestibility. Food Chem..

